# Management of Acute Kidney Injury and Extracorporeal Blood Purification Therapies During the COVID-19 Pandemic: The Italian SIN–SIAARTI Joint Survey (and Recommendations for Clinical Practice)

**DOI:** 10.3389/fmed.2022.850535

**Published:** 2022-04-07

**Authors:** Silvia De Rosa, Marita Marengo, Stefano Romagnoli, Marco Fiorentino, Vito Fanelli, Enrico Fiaccadori, Nicola Brienza, Santo Morabito, Vincenzo Pota, Fabrizio Valente, Giacomo Grasselli, Piergiorgio Messa, Antonino Giarratano, Vincenzo Cantaluppi

**Affiliations:** ^1^Department of Anesthesiology and Intensive Care, International Renal Research Institute of Vicenza, San Bortolo Hospital, Vicenza, Italy; ^2^Nephrology and Dialysis Unit, Azienda Sanitaria Locale (ASL) CN1, Cuneo, Italy; ^3^Section of Anesthesiology, Department of Health Sciences, Intensive Care and Pain Medicine, University of Florence, Florence, Italy; ^4^Nephrology, Dialysis and Transplantation Unit, Department of Emergency and Organ Transplantation, University of Bari, Bari, Italy; ^5^Anaesthesia, Critical Care and Emergency, A.O.U. Department of Surgical Sciences, Città della Salute e della Scienza di Torino, University of Turin, Turin, Italy; ^6^Dipartimento di Medicina e Chirurgia, Università di Parma, UO Nefrologia, Azienda Ospedaliero-Universitaria Parma, Parma, Italy; ^7^Section of Anesthesia and Intensive Care Unit, Department of Interdisciplinary Medicine, University of Bari, Bari, Italy; ^8^UOSD Dialisi, Azienda Ospedaliero-Universitaria Policlinico Umberto I, “Sapienza” Università di Roma, Rome, Italy; ^9^Department of Women, Child and General and Specialized Surgery, University of Campania Luigi Vanvitelli, Naples, Italy; ^10^Ospedale S. Chiara di Trento, USC Nefrologia e Dialisi, Trento, Italy; ^11^Department of Pathophysiology and Transplantation, University of Milan, Milan, Italy; ^12^UOC Nefrologia Dialisi e Trapianto Fondazione IRCCS Ca' Granda Ospedale Maggiore–Policlinico di Milano, Milan, Italy; ^13^Section of Anesthesia, Analgesia, Intensive Care and Emergency, Policlinico Paolo Giaccone, Department of Biopathology and Medical Biotechnologies, University of Palermo, Palermo, Italy; ^14^Nephrology and Kidney Transplantation Unit, Department of Translational Medicine, University of Piemonte Orientale, “Maggiore della Carità” University Hospital, Novara, Italy

**Keywords:** acute kidney injury, COVID-19, critical care, renal replacement therapy, blood purification, surveys and questionnaires

## Abstract

**Background and Aim:**

The novel coronavirus disease 2019 remains challenging. A large number of hospitalized patients are at a high risk of developing AKI. For this reason, we conducted a nationwide survey to assess the incidence and management of AKI in critically ill patients affected by the SARS-CoV-2 infection.

**Methods:**

This is a multicenter, observational, nationwide online survey, involving the Italian Society of Nephrology and the critical care units in Italy, developed in partnership between the scientific societies such as SIN and SIAARTI. Invitations to participate were distributed through emails and social networks. Data were collected for a period of 1 week during the COVID-19 pandemic.

**Results:**

A total of 141 responses were collected in the SIN–SIAARTI survey: 54.6% from intensivists and 44.6% from nephrologists. About 19,000 cases of COVID-19 infection have been recorded in hospitalized patients; among these cases, 7.3% had a confirmed acute kidney injury (AKI), of which 82.2% were managed in ICUs. Only 43% of clinicians routinely used the international KDIGO criteria. Renal replacement therapy (RRT) was performed in 628 patients with continuous techniques used most frequently, and oliguria was the most common indication (74.05%). Early initiation was preferred, and RRT was contraindicated in the case of therapeutic withdrawal or in the presence of severe comorbidities or hemodynamic instability. Regional anticoagulation with citrate was the most common choice. About 41.04% of the interviewed physicians never used extracorporeal blood purification therapies (EBPTs) for inflammatory cytokine or endotoxin removal. Moreover, 4.33% of interviewed clinicians used these techniques only in the presence of AKI, whereas 24.63% adopted them even in the absence of AKI. Nephrologists made more use of EBPT, especially in the presence of AKI. HVHF was never used in 58.54% of respondents, but HCO membranes and adsorbents were used in more than 50% of cases.

**Conclusion:**

This joint SIN–SIAARTI survey at the Italian Society of Nephrology and the critical care units in Italy showed that, during the COVID-19 pandemic, there was an underestimation of AKI based on the “non-use” of common diagnostic criteria, especially by intensivists. Similarly, the use of specific types of RRT and, in particular, blood purification therapies for immune modulation and organ support strongly differed between centers, suggesting the need for the development of standardized clinical guidelines.

## Introduction

Coronavirus disease 2019 (COVID-19), the disease caused by severe acute respiratory syndrome CoV-2 (SARS-CoV-2), is a global pandemic of unprecedented proportions. SARS-CoV-2 is mainly characterized by moderate/severe pneumonia associated with progressive endothelial damage and coagulopathy but may also involve the kidneys. Recent studies suggested that the incidence of acute kidney injury (AKI) during hospitalization in patients with COVID-19 has a wide range and that AKI is associated with a poor prognosis (1–3). COVID-19 patients with AKI had a significantly higher mortality rate of 54.24% and, overall, had 18 times higher risk of death when compared to COVID-19 patients without AKI (4). A systematic review, including 22 retrospective cross-sectional studies with 16,199 patients hospitalized with COVID-19 from January 1 2020 to June 1 2020, demonstrated that AKI was not rare in patients with COVID-19. The incidence of AKI could be associated with age, disease severity, and ethnicity (5). The cause of AKI in patients with COVID-19 is multifactorial, including a direct attack by SARS-CoV-2 (the Consensus Report of the 25th Acute Disease Quality Initiative-ADQI Workgroup) or by hemodynamic instability, microcirculatory dysfunction, tubular cell injury, renal congestion, microvascular thrombi, and endothelial dysfunction (6). The incidence of AKI was 26% in intensive care unit (ICU) patients, and the disease severity was associated with the presence of AKI in patients with COVID-19 (4). The novel coronavirus disease 2019 (COVID-19) is challenging, given a large number of hospitalized patients. Cardiovascular comorbidities are linked to a higher mortality risk. Thus, patients with AKI might represent a frail population at a high risk of poor outcomes and of long-term multiple organ dysfunction or “long COVID.”

Additionally, in the critical care setting, there is evidence of a marked variation in the management of AKI due to both a lack of awareness and a lack of standardization of methods for prevention, early recognition, and intervention (7, 8). Insights into the opinions of ICU physicians regarding COVID-19 management and an adequate application of AKI definition criteria would allow the early recognition and implementation of measures aimed at the prevention of kidney damage. This aims at improving patient management, facilitating uniformity in future studies and helping in the formation of future guidelines. For these reasons, we collected data from a multicenter, observational, nationwide survey among anesthesia intensive care (AIC) and nephrology physicians in order to assess the incidence and management of AKI during the pandemic waves of COVID-19.

## Methods

This is a multicenter, observational, nationwide online survey, involving the Italian Society of Nephrology and critical care units in Italy, developed in partnership between the scientific societies, such as Italian Society of Nephrology (SIN) and Italian Society of Anesthesiology and Critical Care (SIAARTI). A joint committee SIN–SIAARTI formed by six nephrologists and six intensivists, all with clinical and research experience on AKI and CRRT, prepared the survey: SIN and SIAARTI distributed the questionnaire to all their members with a maximum of two reminders. Invitations to participate were distributed through emails and social networks (A link for email registration was disseminated *via* social media including Twitter, Facebook, and Linkedin). A short introduction and a link to the survey were available to share on social media. The online questionnaire (Supplementary Material 1) was available for 1 week.

### Survey Development

The objective of this survey was to provide insights into the opinion of AIC and nephrology physicians regarding the main aspects of AKI in patients with COVID-19: 1) definition and incidence and 2) monitoring and management. We combined these topics into a single questionnaire with three sections to minimize the burden for respondents, since both topics are closely related and would be studied in the same target population.

The questionnaire was built using SurveyMonkey Platinum (SurveyMonkey Inc., San Mateo, CA, USA). The questionnaire consisted of 23 questions in total. All answers could be reviewed and edited until final submission. Information about the survey and its purpose was explained at the beginning of the online survey. Respondents' demographics (including specialty, age, and sex) and the number of beds for COVID 19 in the hospital (wards, semi-intensive care, and intensive care units) were collected in questions 1 to 3. The first section of the questionnaire (questions 4 to 11) focused on the definition of AKI. The second section of the questionnaire (questions 12 to 19) included questions on renal replacement therapy (RRT). The third section of the questionnaire (questions 20 to 23) included topics on extracorporeal blood purification techniques (EBPTs) for the removal of inflammatory mediators.

### Target Population

AIC and nephrology physicians working on patients with COVID-19 during the pandemic waves in Italy were the target population. Both AIC and residents in anesthesia and intensive care practicing in ICU are referred to as intensive care physicians; nephrologists and residents in nephrology are referred to as nephrology physicians. Respondents were instructed to answer questions from the perspective of their standard clinical practice. In addition, they had the opportunity to contact the research team if they had additional questions or if they wanted to receive a summary of the study findings.

### Data Analysis

Data were downloaded as a.csv file and were subsequently stored as an Excel file (Microsoft Corp, Redmond, WA, USA). Responses were included in the analyses if both the demographic questions and at least one question from the second section of the questionnaire were answered.

The exclusion criteria included occupation other than ICU physician or nurse and open-ended questions answered in a different language. Missing data were not inputted. Descriptive statistics, performed using MS Excel, were used to analyze the findings. Continuous variables were summarized descriptively using median and interquartile range, while categorical variables were summarized using counts and percentages.

## Results

### Acute Kidney Injury (AKI)

**Figure 1:**
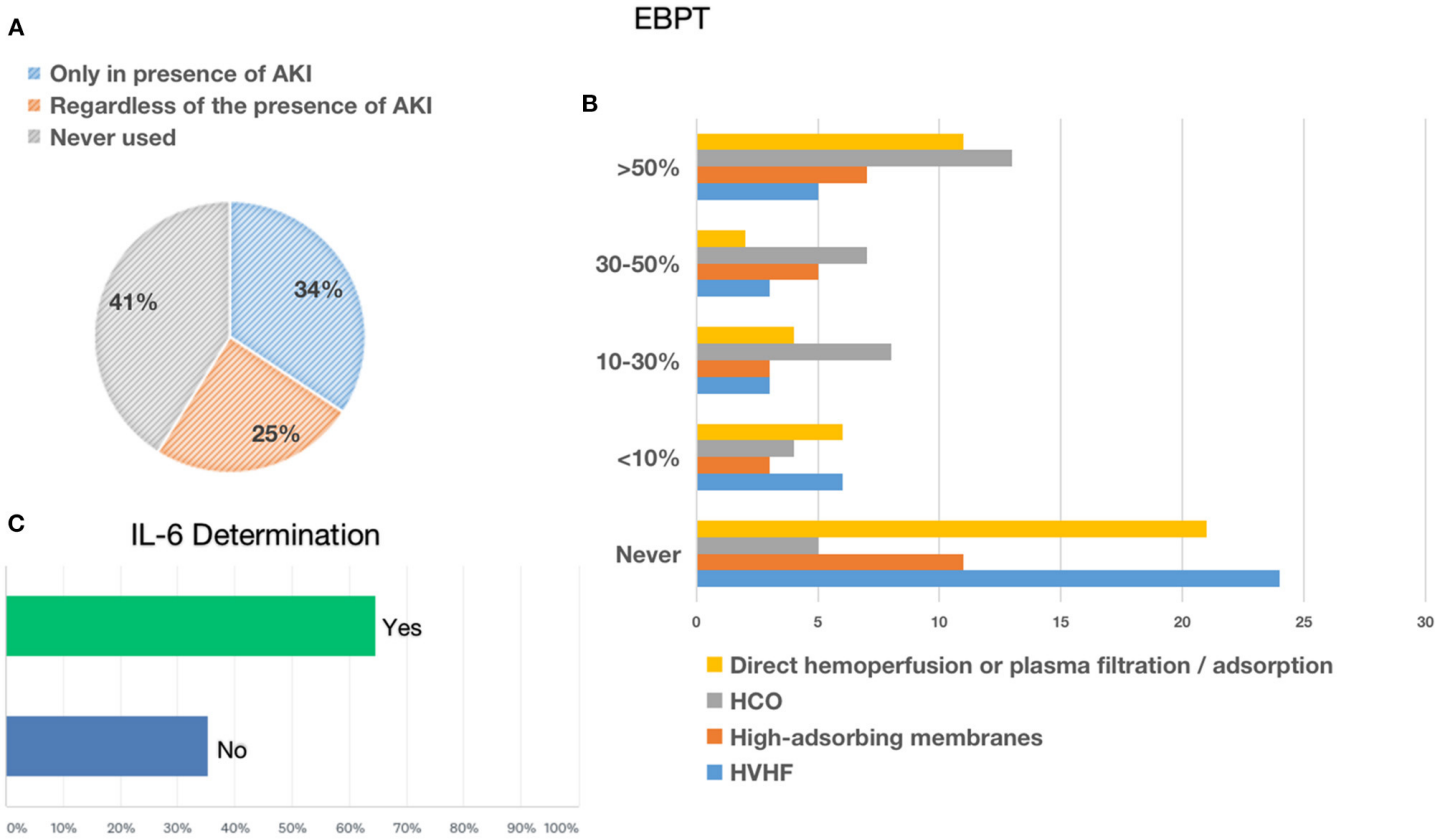
Acute kidney injury. **(A)** Shows the percentage of underestimation of acute kidney injury (AKI) referred only to patients who needed RRT. **(B)** Shows the use of AKI definition criteria (AKIN, KDIGO, RIFLE, etc.) to classify AKI in patients with COVID-19. **(C)** Shows the use of urinalysis or biomarkers for AKI in patients with COVID-19.

About 19,000 cases of COVID-19 infection have been recorded in hospitalized patients; among these, 1,393 cases (7.3%) of AKI were found, of which 1,146 (82.2%) were in ICU. The median of COVID-19–positive hospitalizations with AKI per center was 5, since it appears symmetrical for both the nephrologist and the AIC groups. Both groups agree on the underestimation of AKI (82.7% among nephrologists and 67% among intensivists) (Figure 1). To determine the presence of AKI, only 43% of clinicians routinely used the international criteria (KDIGO, AKIN, and RIFLE) based on serum creatinine and urinary output. This was slightly more commonly used by nephrologists than AICs. Among those who defined the presence of AKI according to KDIGO, 45% of clinicians stratified AKI according to these criteria without specifying the individual classes (Figure 1). On average, five patients from each center who developed AKI were already affected by CKD, a similar finding for both the nephrologist group and the AIC group. Episodes of AKI in solid organ transplant patients diverged between the two groups, as they were 11.6% in the nephrologist group and 2.2% in the AIC group. Of the AKI patients, 628 underwent different types of RRT (similar percentages in the two groups). About 50% of the recognized AKI cases were not directly related to COVID-19 interstitial pneumonia. Overall, 72.4% of clinicians used urinalysis with sediment, a disproportionate percentage among nephrologists (76.7%) and AIC (47.3%). On the other hand, only two nephrological centers used biomarkers (reported use of cystatin-C) compared to 37 of the AICs (reported the use of cystatin-C, NGAL, and Nephrocheck) (Figure 1).

### Renal Replacement Therapies (RRTs)

**Figure 2:**
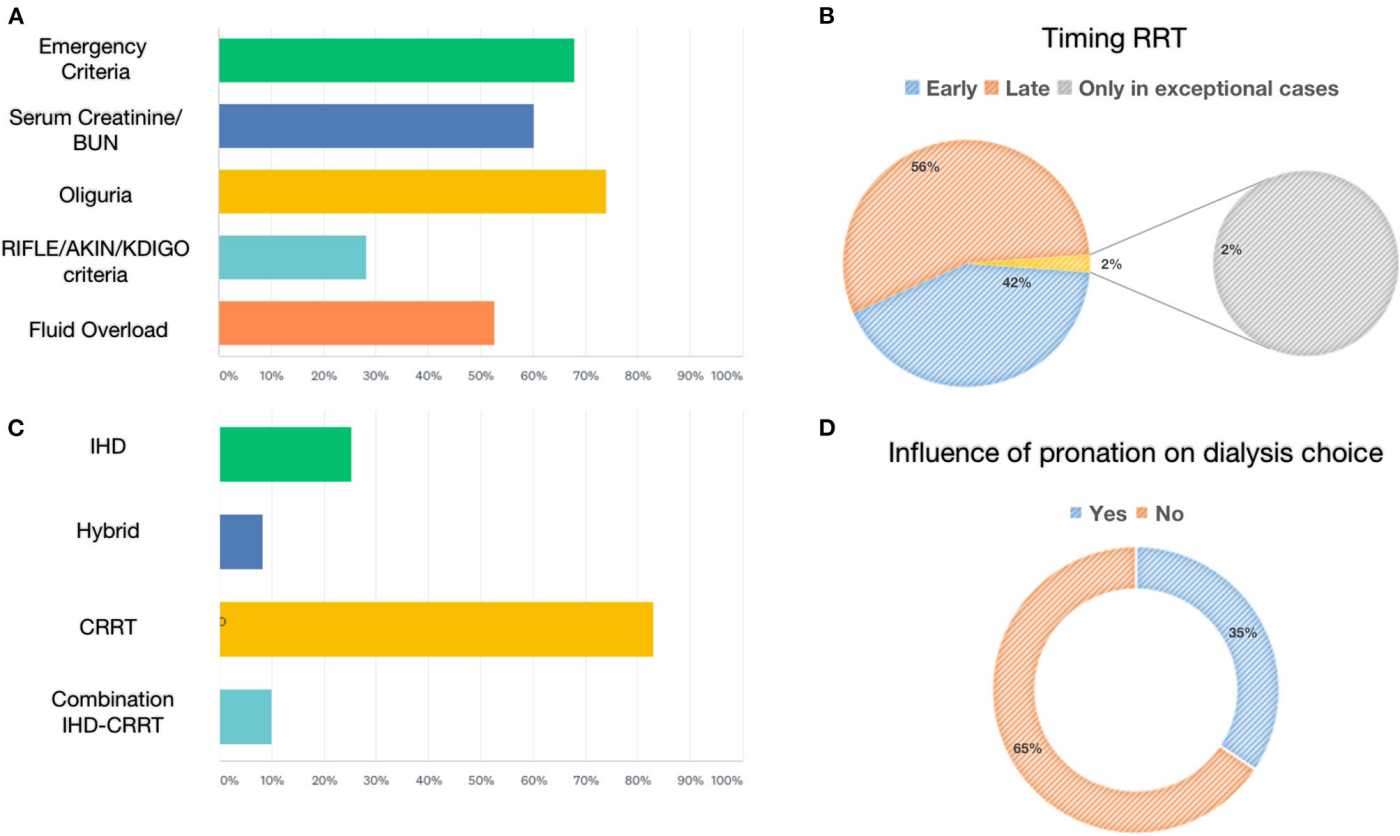
Renal replacement therapies: indication, modality, timing, and prone positioning influence. **(A)** Shows the percentage of parameters used to define the indication to start RRT. **(B)** Shows the percentage of early vs. late initiation of RRT. **(C)** Shows the percentage of RRTs for AKI. **(D)** Shows the percentage influencing the choice of dialytic strategy due to prone positioning.

**Figure 3:**
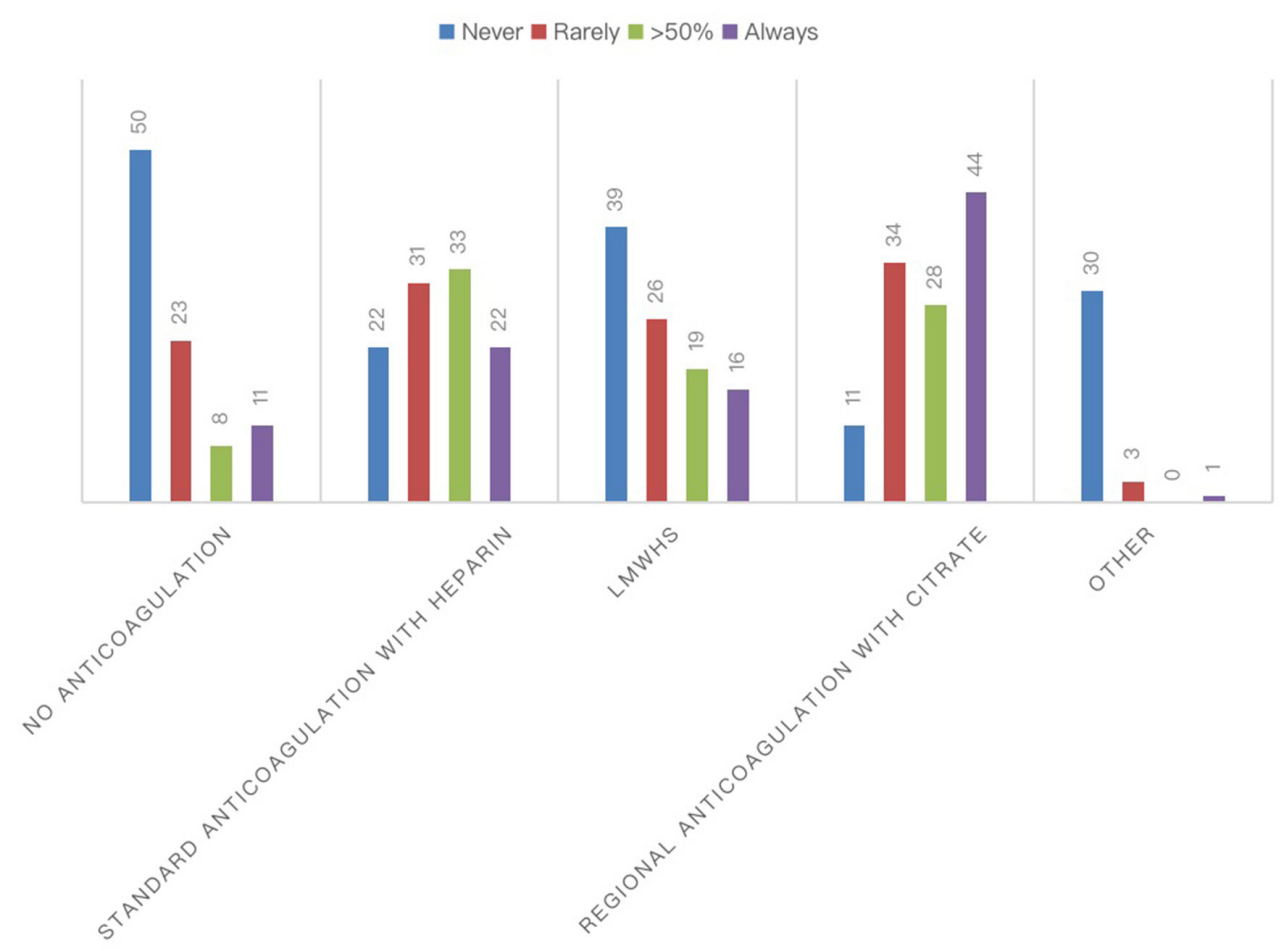
Anticoagulation strategies for RRTs during COVID-19 pandemic. The chart showed the absolute number of different anticoagulation strategies for the extracorporeal circuit in patients with COVID-19.

The most frequently used RRT modalities were continuous techniques in 83.08% (CVVH, CVVHD, or CVVHDF), which represented the absolute majority among AICs (78%) and preferred method among nephrologists (53%), followed by 25.38% receiving intermittent hemodialysis (IHD) and 8.46% prolonged intermittent methods (PIRRT, SLED) (Figure 2). Oliguria was present in 74.05% of cases, and the classic indications to start RRT (hyperkalemia, metabolic acidosis, etc.) were present in 67.94% of the answers. Among 60.31% of cases, the indication on the levels of serum creatinine and urea was given in 52.67% of cases for fluid overload (Figure 2), and in only 28.24% of cases, it was based on the AKI definition criteria such as KDIGO, AKIN, or RIFLE, of which AKIN and RIFLE were mostly used among nephrologists (Figure 2). The main exclusion criteria for the initiation of RRT were as follows: therapeutic withdrawal, severe comorbidities, or hemodynamic instability. Most clinicians preferred early initiation of RRT (73.48%). Another question was whether prone positioning influenced the choice of method for these patients: In two-thirds of cases (65.65%), this did not affect it. The last aspect addressed was related to the anticoagulation strategy adopted in this context (Figure 3). Anticoagulation with unfractionated heparin was used, especially in resuscitation contests, with the following percentages: never, 20.37%; rarely used, 28.7%; in more than 50% of cases, 30.56%; and always, 20.37%. Low molecular weight heparin was used with the following percentages: never used, 39%; rarely, 26%; > 50% of cases, 19%; and always, 16%. Regional anticoagulation with citrate was the most widespread choice among both resuscitators and nephrologists and was adopted with the following percentages: never, 9.4%; rarely used, 29.06%; in more than 50% of cases, and 23.93%; always 37.61%. No anticoagulation was used with the following percentages: never, 54.35%; rarely, 25%; in more than 50% of cases, 8.7%; and always, 11.96%. Finally, other anticoagulation methods, such as bivalirudin and NAO, were used in one of the cases (2 participants), with the following percentages: rarely, 8.82% and always, 2.94%.

### Extracorporeal Blood Purification Therapies

**Figure 4:**
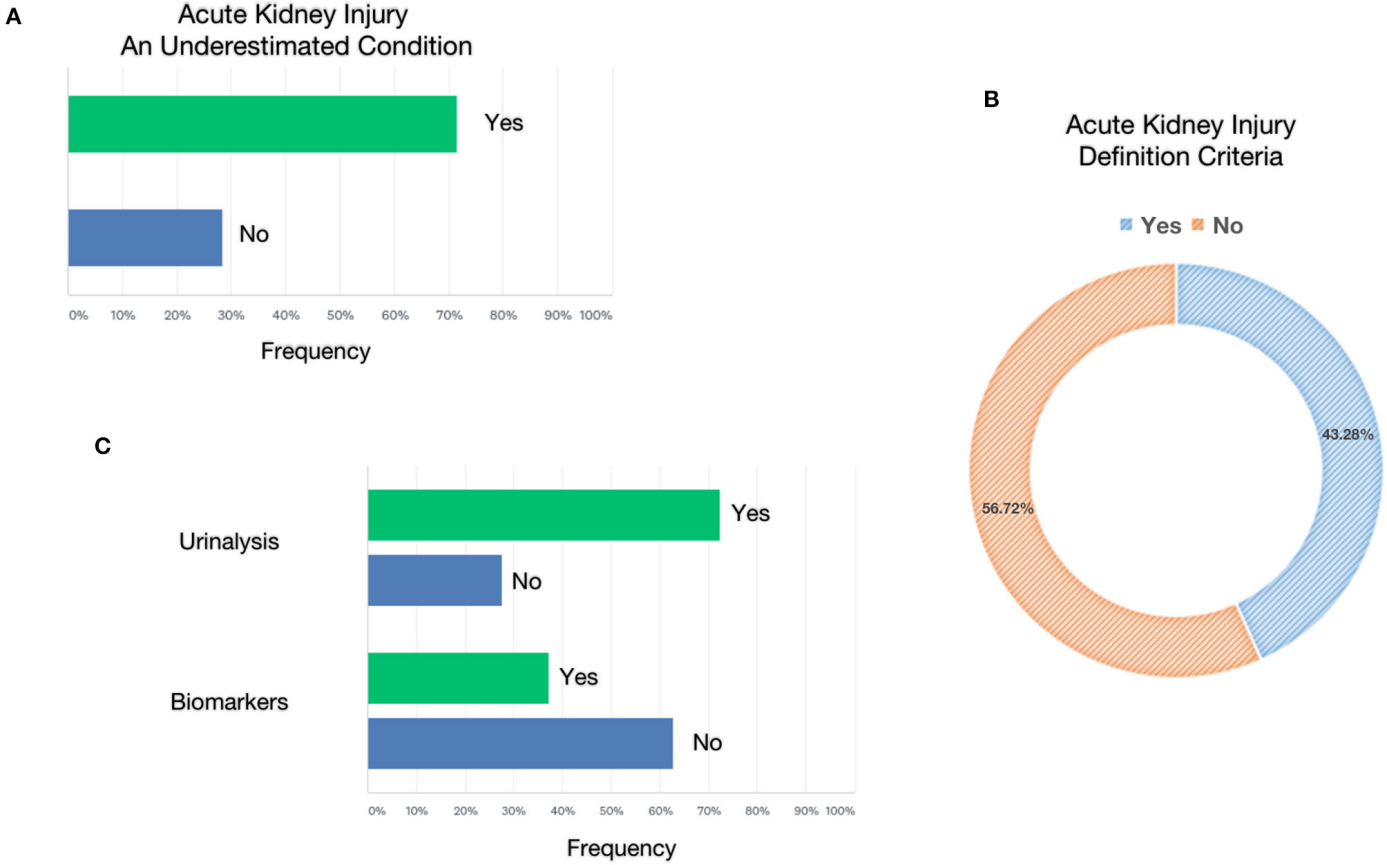
Extracorporeal blood purification therapies**. (A)** Reported the conditions considered to start extracorporeal blood purification therapies. **(B)** Reported the membranes or modalities used in patients with COVID-19. **(C)** Reported the likelihood to test interleukin-6 (IL-6) in enrolled centers.

In recent years, various extracorporeal blood purification therapies have been used for the removal of inflammatory mediators in sepsis. The present survey showed that, in the context of the COVID-19 pandemic in Italy, 41.04% of the interviewed clinicians never used EBPTs, while one-third of them (34.33%) used these techniques only in the presence of AKI; by contrast, 24.63% of clinicians resorted to these techniques even in the absence of AKI (Figure 4). In this context, nephrologists used EBPTs, especially in the presence of AKI. Regarding the different types of EBPTs used, high-volume hemofiltration (HVHF), in absolute terms, was the last choice since it was used by 14.63% in <10% of cases, 7.32% in 10–30% of cases, 7.32% in 30–50%, 12.2% in more than 50% of cases, and never used by 58.54% of the interviewees. High cut-off (HCO) membranes were used by 10.81% of respondents in <10% of cases, 21.62% in 10–30% of cases, 18.92% in 30–50%, 35.14% in more than 50% of cases, and never used by 13.51% of the interviewees. High-adsorbing membranes were used by 10.34% of respondents in <10% of cases, 10.34% in 10–30% of cases, 17.24% in 30–50% of cases, 21.14% in more than 50% of cases, and never used by 37.93% of the interviewees. HCO membranes with high adsorbing properties were the preferred choices of nephrologists who represented the largest number of clinicians who answered questions about EBPT. Other systems on direct hemoperfusion or plasma filtration/adsorption that were equally distributed among resuscitators and nephrologists, that is, 18 participants used CytoSorb® (CytoSorbents Corporation, NJ, USA), three used EMIC-2 (Fresenius, Bad Homburg, Germany), three used Toraymixin (Toray Industries, Tokyo, Japan), two used Theranova (Gambro Dialysatoren, Hechingen, Germany, a subsidiary of Baxter International), two used Oxiris (Baxter, Meyzieu, France), two used HA330 (Zhuhai Lizhu Group of Biological Material Co, Ltd., China), and one used HFR Supra (Bellco, Mirandola, Italy), were used by 13.64% of the respondents in <10% of cases, 9.09% in 10–30% of cases, 4.55% in 30–50% of cases, 25% in more than 50% of cases, and never used by 47.73% of the interviewees. Finally, 64.71% of the interviewees stated that they can measure the serum levels of IL-6 in their center (Figure 4).

### Survey Respondents

A total of 141 physicians filled in the online SIN–SIAARTI COVID-19 survey in Italy: 77 (54.6%) AIC, 63 (44.6%) nephrologists, and only one (0.8%) from another specialty whom we did not consider in our descriptive analysis. Baseline characteristics are reported in Table 1. Among those who compiled the survey responses, the work was carried out mainly by specialists with more than 15 years of experience both for the AIC group (35%) and for nephrologists (76%). In both groups, men were preponderant. A median value of 80 cases (maximum 2,500 cases) of COVID-19 infection have been admitted in hospital; among these, five AKI cases were found, of which four were observed in ICU. The average number of beds for patients with COVID-19 in individual hospitals showed a significant deviation between the data recorded by the AIC compared to the nephrologists as regards the ordinary hospitalization (88 beds vs. 130), while in the semi-intensive unit and in the ICU, the data were more homogeneous (16 vs. 25 and 22 vs. 23, respectively). As reported in the “Methods” and “Results” sections, the survey was divided into three main parts: A) acute kidney injury (AKI); B) renal replacement therapies (RRTs); C) extracorporeal blood purification therapies (EBPTs) for the removal of inflammatory mediators.

**Table 1:** Baseline characteristics.

**Variable**	**Total**	**Anesthesiologists-intensivists**	**Nephrologists**
	***n* = 140**	***n* = 77**	***n* = 63**
Male, n (%)	87 (62)	45 (45)	42 (43)
**Years of experience, n (%)**			
<5	12 (6)	9 (11)	4 (7)
<10	14 (10)	10 (10)	4 (6)
<15	15 (11)	8 (13)	7 (11)
> 15	90 (64)	42 (54)	48 (76)
Residents	9 (9)	9 (12)	0 (0)
Hospital COVID-19 beds	80 (0–650)	70 (0–400)	120 (0–650)
Semi- intensive COVID-19 beds	15 (0–134)	10 (0–90)	20 (0–134)
Intensive COVID-19 beds	17 (0–400)	16 (0–150)	20 (0–400)
**Employed in, n (%)**			
Abruzzo	2 (2)	2 (3)	0 (0)
Basilicata	1 (1)	1 (1)	0 (0)
Calabria	6 (4)	6 (8)	0 (0)
Campania	7 (5)	5 (6)	2 (3)
Città del Vaticano	0 (0)	0 (0)	0 (0)
Emilia-Romagna	10 (7)	5 (6)	5 (8)
Friuli-Venezia Giulia	4 (3)	3 (4)	1 (2)
Lazio	8 (5)	4 (5)	4 (6)
Liguria	2 (2)	1 (1)	1 (2)
Lombardia	23 (16)	17 (22)	6 (0.9)
Marche	3(2)	2 (3)	1 (2)
Molise	0 (0)	0 (0)	0 (0)
Piemonte	24 (17)	9 (12)	15 (24)
Puglia	9 (6)	3 (4)	6 (9)
Repubblica di San Marino	0 (0)	0 (0)	0 (0)
Sardegna	3 (2)	1 (1)	2 (3)
Sicilia	12 (9)	6 (8)	6 (10)
Toscana	9 (6)	2 (3)	7 (11)
Trentino-Alto Adige	4 (3)	2 (3)	2 (3)
Umbria	1 (1)	1 (1)	0 (0)
Valle d'Aosta	2 (2)	1 (1)	1(2)
Veneto	10 (7)	6 (8)	4 (6)
COVID-19 patients admitted in Hospital	80 (0–2,500)	69 (1–2,000)	120 (0–2,500)
COVID-19 patients with AKI	5 (0–100)	5 (0–100)	5 (0–40)
COVID-19 ICU patients with AKI	4 (0–90)	4 (0–90)	4 (0–88)

## Discussion

In this joint SIN–SIAARTI survey, we uncovered unexpected variations in AKI management and RRT practice, including the use of EBPTs among intensive care and nephrology physicians in Italy during the COVID-19 pandemic. These dissimilarities might be associated with different diagnostic and therapeutic approaches including the use of diverse types of extracorporeal therapies that should be structured within precise clinical guidelines.

AKI has been recognized as the most frequent organ dysfunction after respiratory failure following SARS-CoV-2 infection. During the pandemic, AKI epidemiology varied across different geographical areas and with the criteria adopted for the definition of kidney dysfunction (9). AKI is more frequent in the presence of comorbidities such as diabetes, hypertension, obesity, or pre-existent chronic kidney disease (CKD) of different grades based on the reduction of glomerular filtration rate (GFR) and of renal functional reserve (10). However, the reported differences in AKI incidence may be related to whether clinicians used international scoring systems such as KDIGO. Of note, although both nephrologists and AICs stated a low COVID-19–positive hospitalization rate with AKI, a high percentage of physicians agreed on the underestimation of AKI during hospitalization. Moreover, the percentage of accurate clinical diagnosis guided by the standard criteria based on changes in serum creatinine and urinalysis was lower among AICs compared to biomarkers. About 50% of recognized AKI episodes were not directly related to COVID-19 interstitial pneumonia. Despite the acceptance of standardization in the definition of AKI, clinicians routinely underdiagnose it and fail to appreciate that it is associated with considerable morbidity and mortality (11). A lack of awareness of the importance of early recognition and treatment among healthcare team members and the heterogeneity of approaches within the healthcare teams assessing the patient remain a major challenge, particularly in the intensive care setting. AKI remains a clinical diagnosis, and medical judgment is necessary to apply diagnostic criteria and to evaluate the changing status of the patient. Independent from the pandemic waves, AKI within the ICU seems to be underestimated due to the lack of application of standardized international criteria of classification such as KDIGO, AKIN, or RIFLE. New classifications including the use of urinary biomarkers of tubular injury have facilitated the understanding of AKI incidence and its impact, but they are not always well aligned with AKI pathophysiology and everyday clinical practice. On the basis of the results of this survey, our future goal is to build AKI awareness programs to improve early recognition and management involving inclusive interdisciplinary collaboration, to discuss the ongoing need to change some of our current AKI paradigms, and to develop diagnostic methods and provide specific recommendations to improve AKI recognition and care. Moreover, considering the assessment of kidney function, routine urinalysis gives an insight into the renal pathology of the patient. Recent data showed that urinalysis, as a simple test, can be used to predict the development of AKI and mortality and may be used for risk stratification of patients with COVID-19, especially in low-resource settings (12). Timely diagnostic methods using evolving biomarkers raise the prospect of detection of kidney damage before the onset of irreversible loss of function, but they still remain under investigation. There is considerable variation in the RRT decision (13). Recent studies demonstrated that urinary biomarkers are associated with adverse kidney outcomes in patients hospitalized with COVID-19, and this kidney injury may be responsive to treatment (13). Based on this evidence, biomarkers may provide valuable information to monitor kidney disease progression and recovery (14). Moreover, urinary biomarkers reflect the pathogenic mechanisms of tissue damage, and their expanded use may lead to an early therapeutic intervention to prevent AKI.

Several studies clearly showed that different direct and indirect pathogenic mechanisms are involved in COVID-associated AKI; SARS-CoV-2 can be internalized in endothelial cells, podocytes, and tubular epithelial cells, and the presence of viral genetic material in the kidney has been associated with the development of AKI with a worse outcome (15). SARS-CoV-2 evokes an inflammatory response that can enhance tissue injury and trigger coagulation and complement cascades, with pathogenic mechanisms between the lung and the kidney (16). Moreover, other causes and typical clinical features of critically ill patients including organ cross talk, invasive mechanical ventilation, fluid overload, nephrotoxins, and superimposed bacterial infections play a key role in the development of COVID-associated AKI (8).

The increased demand for RRT has been recognized as another significant feature of the COVID-19 pandemic; on the one hand, the augmented incidence of infection in patients with chronic hemodialysis admitted to semi-intensive or intensive care units led to an increased need for dialysis machines in these acute settings. On the other hand, several studies showed an increased incidence of AKI requiring RRT in critically ill patients with COVID-19, with a further increased demand for dialysis monitors, thus favoring intermittent or prolonged rather than continuous therapies (9). The SIN–SIAARTI survey showed that a majority of AICs started RRT in the case of oliguria, followed by classic indications of urgency. This is in contrast with the evidence previously reported in the literature: In critically ill patients with AKI, there is no added benefit from early initiation of RRT. Delaying the initiation of RRT with close monitoring and initiating RRT for emergent indications should be the accepted criterion in critical care nephrology as reported in a recent updated meta-analysis (17).

Anticoagulation was not used by the majority of physicians followed by regional anticoagulation and then unfractionated heparin. This is in line with the previous meta-analyses that have evaluated the efficacy and safety of regional citrate vs. heparin anticoagulation (18–20). Because of the unprecedented increase in critically ill patients with COVID-19, the capacity to provide CRRT for AKI may quickly be overwhelmed (21). Exacerbating this resource crunch is the hypercoagulability observed in patients with COVID-19. Frequent CRRT circuit clotting leads to blood loss and wastage of already overextended resources, and the need for troubleshooting increases the exposure of healthcare providers to the infected patients (22). However, the majority of clinicians separated circuit anticoagulation from the need for specific therapies aimed at inhibiting the triggering of coagulation typical for patients with COVID-19.

The simultaneous activation of inflammation, coagulation, and complement described a clinical scenario not different from that observed in sepsis-associated AKI; for this reason, the use of EBPTs to either selectively or not selectively remove PAMPs and DAMPs from the bloodstream has been proposed. However, during the COVID-19 pandemic in Italy, 41.04% of the interviewed clinicians never used EBPT, whereas it was particularly adopted by nephrologists in the presence of AKI. Unfortunately, this may be linked to the fact that, in Italy, blood purification therapies are entrusted either to the nephrologist or to the intensivist but rarely to a shared group with mixed specialties. Furthermore, the culture on blood purification therapies is scarce, and renal replacement treatments are often followed according to the clinical practices of the center without well-defined protocols and are aimed at personalization of the treatments. In this scenario, the pandemic has contributed to highlighting this criticality in an important way. Regarding the different types of EBPTs used, HVHF was less frequently used, while HCO and high-adsorption membranes were the preferred choices of nephrologists who predominantly answered questions about EBPT. This inter-physician variation may possibly be explained by the respondents' estimation of the perceived probability of benefits with the use of RRT or EBPT in patients with COVID-19.

We also asked about the possibility of testing interleukin-6 (IL-6) and whether the physicians know the mean serum concentration of this cytokine. As previously confirmed in a recent systematic review and meta-analysis, the serum levels of IL-6 are significantly elevated in the setting of complicated COVID-19 disease, and increased IL-6 levels are significantly associated with adverse clinical outcomes. In addition, ongoing controlled clinical studies aim to elucidate the role of immunomodulation therapies in the most severe forms of COVID-19 (23).

## Strengths and Limitations of This Study

Online surveys enable the collection of anonymized information and facilitate the collection of data from a wide range of patients regardless of their residency. The survey was disseminated through social media to reach the general population. The survey had several “other” options where physicians could give a more detailed explanation of their answers in addition to the multiple-choice answers. The advertisement through social media could have caused selection bias as the physician who does not use social media could not have taken the survey. Our study population might not be a good reflection of the general population of intensivists and nephrologists.

## Conclusion

During the COVID-19 pandemic, there was an underestimation of AKI in Italy based on the “non-use” of established diagnostic criteria such as KDIGO guidelines. The analysis of urinary sediment and AKI biomarkers was not diffused. Moreover, the management of RRTs and EBPTs was very heterogeneous. Future national clinical studies are recommended to investigate the role of a collaborative approach between nephrologists and intensivists on patient outcomes and other controversial topics on AKI, RRTs, and EBPTs in patients with COVID-19. The joint committee SIN–SIAARTI may represent a useful tool to promote inter-society collaboration to study AKI in the critical care setting.

## Data Availability Statement

The data analyzed in this study is subject to the following licenses/restrictions: Data are property of SIAARTI and SIN societies. Requests to access these datasets should be directed to cristina.cacciagrano@siaarti.it.

## Author Contributions

SD and VC conceived the study, collected and analyzed data, and drafted the manuscript. MM and SR analyzed data and contributed to the writing of the manuscript. MF, VF, EF, NB, SM, VP, FV, and GG contributed to the writing of the manuscript. PM and AG revised the manuscript. All authors read and approved the final manuscript.

## Funding

SIAARTI and SIN society supported open access publication fees.

## Conflict of Interest

The authors declare that the research was conducted in the absence of any commercial or financial relationships that could be construed as a potential conflict of interest.

## Publisher's Note

All claims expressed in this article are solely those of the authors and do not necessarily represent those of their affiliated organizations, or those of the publisher, the editors and the reviewers. Any product that may be evaluated in this article, or claim that may be made by its manufacturer, is not guaranteed or endorsed by the publisher.
