# Supplementary material for: Management of Acute Kidney Injury and Extracorporeal Blood Purification Therapies During the COVID-19 Pandemic: The Italian SIN–SIAARTI Joint Survey (and Recommendations for Clinical Practice)
Source: Front Med (Lausanne). 2022 Apr 7;9:850535. doi: 10.3389/fmed.2022.850535 (PMC9021595; doi:10.3389/fmed.2022.850535)
Supplement: Supplementary file 1 [file Data_Sheet_1.pdf]

Supplemental material 1. Adapted English version of the online survey

SIN-SIAARTI Survey:
Acute Kidney Injury (AKI) and Blood Purification Therapy in COVID-19

Dear Collegue,
the COVID-19 pandemic that our country is facing in recent weeks has completely changed the structure of our hospitals, resulting in an overload of work for medical and nursing staff, especially in intensive care. However, we believe that the hallmark of a Scientific Society is also to collect and share the data and experiences of this difficult moment to analyze them rigorously and then make them available to the medical community in a short time. For this reason, we would like to involve you in the preparation of a consensus document on management procedures on AKI and CRRT in COVID-19, in order to photograph epidemiological data and clinical practice on the national territory and to potentially provide a guideline for the centers and countries reached later by SARS-CoV-2. As part of the SIN-SIAARTI Joint Commission, we are committed to formulating some questions collected in the following Survey that will allow us to understand which are the most widespread local clinical practices and to formulate potential shared statements in the near future.We therefore ask you to answer the following short questions in relation to your Center's activity in the field of AKI and renal replacement therapies during the last few weeks. It is good that this can also concern a moment of sharing between nephrologists and resuscitators to get even closer on these issues. We would be grateful if you could answer your questions by ..... (in my opinion 3-5 days max from receiving mail) We thank you for the attention and time you want to dedicate to this initiative.
Best regards,

The SIN-SIAARTICommission

Some tips before starting:

1) Your participation because it is very important: you must be as accurate as possible in filling out the questionnaire.
2) Do not consider the questionnaire as a test: it is simply a survey on the real world of clinical practice.

We need to know your actions in day-to-day reality (not what you should do if you could, or what you think is the best answer, but what happens in your department).

3) If not specifically specified, select only one answer. If you think more than one option may be applicable to your clinical practice, try to select the most common choice. If you are unsure or don't know the answer, it is best not to select any option (unless the "don't know" option is available).

Section I - GENERAL INFORMATION

☐ Anaesthesiologist and Intensivist

☐ Nephrologist

☐ Other: Specify……………..

COVID19 Cohort:

- **Ward:**
- **Semi-intensive Unit:**
- **Intensive Unit:**

1. Age: ___ years
2. Sex: ☐ Man ☐ Woman
3. How many beds were allocated for COVID-19 patients in your hospital ?
   (In the case of semi-intensive units, intensive or dialysis units , you should indicate the total of beds)

N.COVID Beds (Hospital) ☐ <100 ☐100-300 ☐ 300-600 ☐ 600-1000 ☐ >1000
N.COVID Semi-intensive Beds ☐ <10 ☐ 10-20 ☐ 20-50 ☐ >50
N.COVID Intensive Beds ☐ <10 ☐ 10-20 ☐ 20-50 ☐ >50

Section II - Acute Kidney Injury (AKI)

4. How many COVID-19 cases are admitted in total in your hospital ? (Open question)
5. How many COVID-19 cases managed in your hospital presented AKI? And how many in intensive care unit?(Open question)
6. Do you think that the incidence of AKI are underestimated because it is referred only to patients that needed RRT? (Yes/No)
7. Did you used the AKI Definition Criteria (AKIN, KDIGO, RIFLE, etc) to classify AKI in COVID-19 patients? (Yes/No). If yes, do you know the percentage of patients in different stages (KDIGO 1,2,3)?
8. How many COVID-19 patients admitted in your center presented a previous chronic kidney disease (CKD) based on GFR levels? (Open question)
9. How many COVID-19 patients who developed AKI are solid organ transplant recipients?(Open question)

10. Did you performed Urinalysis or Biomarkers for AKI in COVID-19 patients? (only one answer is requested)

☐ Urinalysis

☐ Biomarkers, if yes indicate which one:...............

11. Which is the percentage of AKI not related to SARS-COV-2 pneumonia in COVID 19 patients (i.e.super-infection/bacterial sepsis, pre-renal causes, nephrotoxicity, etc) ?

Section III - Renal replacement therapy (RRT)

12. How many COVID-19 patients needed RRT for AKI in your hospital? (Open question. Indicate the percentage)
13. Which RRT your equipe is able to perform in COVID-19 patient with AKI ? (Indicate all techniques used)

    ☐ Intermittent Hemodialysis (IHD)

    ☐ Hybrid Therapy (SLEDD/EDD)

    ☐ CRRT (CVVH/CVVHD/CVVHDF)

    ☐ Combination Intermittent/Continuous

    ☐ Other (Specify):
14. Which of the following parameters you used to define the indication to start RRT in COVID-19 patients (if necessary, select also more options)

    ☐ Emergency Criteria (Hyperkalemia, metabolic acidosis,etc.)

    ☐ Urea and/or Serum Creatinine values

    ☐ Oliguria (assessment based on diuresis ml/kg/h)

    ☐ RIFLE, AKIN o KDIGO classification

    ☐ Volume Overload
15. In your center, in COVID-19 patients with AKI you prefer to start RRT (if needed, select more option):

    ☐ Early

    ☐ Late

    Only in selected cases: ....
16. Which are the criteria in your center to exclude the start of RRT? (Open question)
17. Which are the more commonly used drugs in COVID-19 patients with AKI and RRT?(Open question)
18. In your center, prone positioning needed in COVID-19 patients influences the choice of dialytic strategy? (Open question)
19. Which is the prevalence of different anticoagulation techniques of the extracorporeal circuit in COVID-19 patients admitted in your center?

✓ No anticoagulation

Never Rarely >50% Always

✓ Standard anticoagulation with heparin

Never Rarely >50% Always

✓ Heparin with low molecular weight

Never Rarely >50% Always

✓ Regional anticoagulation with calcium-citrate

Never Rarely >50% Always, in absence of contraindication

✓ Other (specify) ....

Never Rarely >50% Always

Section IV - Extracorporeal blood purification therapies

20. Which of the following conditions did you use blood purifications therapy in COVID-19 patients? (if necessary, select more options)

☐ Only in presence of AKI

☐ Independent by presence of AKI

☐ Never used

21. Which of the following membranes or modalities you used in COVID-19 patients? (if necessary, select more options)

☐ High Volume Hemofiltration (HVHF/Pulse HVHF)

Never <10% 10-30% 30-50% >50%

☐ Membranes ad elevated adsorption

Never <10% 10-30% 30-50% >50%

☐ High Cut Off Membrane

Never <10% 10-30% 30-50% >50%

☐ Hemoperfusion or plasma absorption/adsorption

Never <10% 10-30% 30-50% >50%

22. In case of positive answer to previous question, which are the Hemoperfusion or plasma absorption/adsorption that you used? (Open question)

23. Do you have the possibility to test Interleukin-6 (IL-6)? (Yes/No)

If Yes, do you know the mean plasma concentration? (Open question)
